# Bioinformatics identification and pharmacological validation of *Kcnn3*/K_Ca_2 channels as a mediator of negative affective behaviors and excessive alcohol drinking in mice

**DOI:** 10.1038/s41398-020-01099-4

**Published:** 2020-11-27

**Authors:** Audrey E. Padula, Jennifer A. Rinker, Marcelo F. Lopez, Megan K. Mulligan, Robert W. Williams, Howard C. Becker, Patrick J. Mulholland

**Affiliations:** 1 Department of Neuroscience, Medical University of South Carolina, Charleston, SC 29425 USA; 2 Charleston Alcohol Research Center, Medical University of South Carolina, Charleston, SC 29425 USA; 3 Department of Psychiatry & Behavioral Sciences, Medical University of South Carolina, Charleston, SC 29425 USA; 4 Department of Genetics, Genomics and Informatics, University of Tennessee Health Science Center, Memphis, Tennessee 38163 USA

**Keywords:** Neuroscience, Genetics

## Abstract

Mood disorders are often comorbid with alcohol use disorder (AUD) and play a considerable role in the development and maintenance of alcohol dependence and relapse. Because of this high comorbidity, it is necessary to determine shared and unique genetic factors driving heavy drinking and negative affective behaviors. In order to identify novel pharmacogenetic targets, a bioinformatics analysis was used to quantify the expression of amygdala K^+^ channel genes that covary with anxiety-related phenotypes in the well-phenotyped and fully sequenced family of BXD strains. We used a model of stress-induced escalation of drinking in alcohol-dependent mice to measure negative affective behaviors during abstinence. A pharmacological approach was used to validate the key bioinformatics findings in alcohol-dependent, stressed mice. Amygdalar expression of *Kcnn3* correlated significantly with 40 anxiety-associated phenotypes. Further examination of *Kcnn3* expression revealed a strong eigentrait for anxiety-like behaviors and negative correlations with binge-like and voluntary alcohol drinking. Mice treated with chronic intermittent alcohol exposure and repeated swim stress consumed more alcohol in their home cages and showed hypophagia on the novelty-suppressed feeding test during abstinence. Pharmacologically targeting *Kcnn* gene products with the K_Ca_2 (SK) channel-positive modulator 1-EBIO decreased drinking and reduced feeding latency in alcohol-dependent, stressed mice. Collectively, these validation studies provide central nervous system links into the covariance of stress, negative affective behaviors, and AUD in the BXD strains. Further, the bioinformatics discovery tool is effective in identifying promising targets (i.e., K_Ca_2 channels) for treating alcohol dependence exacerbated by comorbid mood disorders.

## Introduction

Alcohol use disorder (AUD) is a devastating disease that causes significant disruption of brain function and behavior. While environmental factors and drinking history are obviously critical, heritability estimates for AUD in many modern societies are upwards of 60%. Defining and understanding genetic variants among individuals in regards to AUD resistance, susceptibility, and relapse risk will provide key data to develop more personalized prevention and treatment plans^1,2^. AUD is frequently comorbid with affective mood disorders^3,4^ and this fact often limits available treatment interventions^5^. The negative affective disturbances that tend to develop during abstinence from alcohol (ethanol) drinking can drive relapse-like behaviors in both humans and rodent models^6–12^. This is complicated further by preclinical and clinical evidence that links increased drinking with the use of drugs such as selective serotonin reuptake inhibitors commonly used to treat depression and anxiety^13,14^. Moreover, alcohol consumption increases depressive symptoms^15^. Because of these limitations and the fact that current Food and Drug Administration-approved pharmacotherapies for AUD do not target mood disorders, it is essential to identify new targets that treat both excessive alcohol drinking and negative affective states. Despite increased efforts to identify mechanisms that drive excessive drinking and negative affective disturbances and the push to move toward a personalized medicine approach, treatment options for individuals with AUD are still limited.

One class of drugs that has shown promise in both preclinical and clinical studies are those that target potassium (K^+^) channels, which represent a large, diverse class of ion channels that modulate many aspects of the biophysical membrane properties of neurons and glia and ultimately influence activity of neural circuits. Recently, a number of K^+^ channels have been implicated in neuropsychiatric conditions, including AUD and mood disorders. Chronic alcohol exposure produced adaptations in K^+^ channels that influenced intrinsic excitability and excessive intake^16^. Pharmacological targeting of K^+^ channels reduced alcohol self-administration, enhanced extinction of alcohol-seeking behavior, and decreased intake in rodents with a heavy drinking phenotype^2,16–18^. Knockdown of *Kcnk9* in monoaminergic neurons reduced negative affective behaviors in mice^19^. In a social defeat model of depression, mice that were resilient to depressive-like behaviors had higher expression of *Kcnq3* in the ventral tegmental area (VTA) and increased K^+^ channel currents in VTA dopaminergic (DA) neurons^20,21^. Further, Friedman and colleagues^22^ demonstrated that retigabine—a K_V_7 channel-positive modulator—restored normal firing patterns of VTA neurons and prevented the development of depression-associated behaviors. In addition, these authors reported that overexpression of *Kcnq* channels in VTA DA neurons normalized neuronal hyperactivity and depression-like behaviors in susceptible mice. In rats, Kang and colleagues confirmed that retigabine microinfusion into the lateral habenula reduced anxiety-like behaviors during alcohol withdrawal^23^. Additional K^+^ channels, such as K_Ca_2, GIRK1, and K_V_1.3, influenced anxiety-related behaviors^24–28^. These promising findings have motivated our genetic studies on the role of K^+^ channels as a factor linking negative affective behaviors and excessive drinking.

Stressful life events can precipitate the onset of major depressive disorder and are a major risk factor for developing mood disorders^29,30^. Furthermore, the relationship between stress and mood disorders is influenced by gene–environment interactions^31,32^. Evidence demonstrates that the basolateral amygdala (BLA) is an important structure for the intersection of anxiety-like behaviors and excessive alcohol consumption^33–35^. In this study, we used a deep phenome and gene expression data set acquired for many strains of the BXD family of mice^36^ to identify K^+^ channel genes in the BLA that are associated with anxiety-like behaviors. BXD RI strains are generated by intercrossing C57BL/6J and DBA/2J inbred parental strains and their isogenic progeny to generate recombinant strains that are then inbred to produce stable RI lines^37^. The BXD panel has been fully genotyped and sequenced, expression profiling has been completed for numerous brain regions, and >7500 behavioral, morphological, and pharmacological traits have been measured across this population. Thus BXD RI strains are well suited for systems genetics analysis of behavioral traits and are a powerful resource to study the genetic diversity of anxiety- and alcohol-related phenotypes^18,38–40^. Once gene targets were identified, we determined whether expression of the top target (i.e., *Kcnn3* that encodes K_Ca_2.3 or SK3 channels) co-varied with voluntary alcohol drinking. Next, we evaluated the extent to which chronic alcohol and stress co-exposure produces excessive drinking and negative affective behaviors. Because we identified a promising candidate with correlations between anxiety-like behaviors and alcohol consumption, we used a systemic pharmacological approach to validate a key role of K_Ca_2 channels in jointly influencing aberrant behaviors in a model useful for studying alcohol–stress interactions.

## Materials and methods

### Bioinformatics analysis

Spearman correlations between BLA K^+^ channel expression and anxiety-like phenotypes that reached *p* < 0.01 were calculated in GeneNetwork. For the correlational analyses, there were 91 probe sets targeting 88 unique K^+^ channel genes in the INIA Amygdala Cohort Affy MoGene 1.0 ST (Mar11) RMA Database and 302 anxiety-like phenotypes from the BXD Published Phenotypes Database in male and female BXD RI strains. Next, a hypergeometric enrichment analysis was performed to determine whether there was over-representation of significant correlations between the expression of each K^+^ channel gene in the BLA and 302 anxiety-like phenotypes. All data sets used for this analysis are available in the GeneNetwork database^41^. A Bonferroni correction was used to determine significance for the enrichment analysis. *Kcnn3* expression in the BLA (GeneNetwork Record ID 10493555) was also correlated with phenotypes related to alcohol consumption.

### Genetic mapping

To identify genomic loci that control the variability in the network of the top K^+^ channel gene (i.e., *Kcnn3*), an eigentrait (the first principal component from principal components analysis) was computed. To avoid over-sampling of traits containing multiple behavioral measurements of the same phenotype, traits were separated into seven similar activity- or anxiety-like phenotypes, and the trait from these seven phenotypes with the highest correlation with *Kcnn3* expression was selected for further analysis (Supplemental Table 1). In addition, only behavioral traits with sample size of ≥56 strains were included in the eigentrait analysis. The *Kcnn3* eigentrait was mapped to regions of the genome containing gene variants that modulate trait expression using a liner mixed model in the R/qtl2 add-on package^42^ for the statistical software R. Genome-wide significance was established based on 1000 permutations of the data.

### Animals

Adult, male C57BL/6J mice (Jackson Laboratory, Bar Harbor, ME; *n* = 382) were 8–10 weeks of age at the start of each experiment and were individually housed in a temperature- and humidity-controlled vivarium. Mice were kept on a 12-h reverse light/dark cycle with ad libitum access to food (Harlan Teklad Diet 2918) and water with the exception of when mice were tested on the novelty-suppressed feeding test (NSFT). All mice were treated in accordance with the NIH Guide for the Care and Use of Laboratory Animals under protocols approved by the Institutional Animal Care and Use Committee at the Medical University of South Carolina.

### Alcohol and stress models

Mice were treated with a chronic intermittent ethanol (CIE) vapor inhalation and forced swim stress (FSS) interaction model that produces increased home cage alcohol drinking in the stressed, dependent mice following previously reported methods^43–46^. Baseline drinking was established by allowing mice to consume alcohol in their home cage using a standard two-bottle choice (2BC; 15% ethanol (v/v) vs water) limited-access (2 h) protocol for 4–6 weeks. These mice were pseudo-randomly divided into air or alcohol vapor groups such that alcohol intake was equated between conditions, and then underwent two cycles of CIE vapor or air (control group) exposure in inhalation chambers, alternated with two weekly home cage drinking “test” sessions^40,47,48^. Air or CIE exposure was delivered 16 h/day for 4 consecutive days followed by 72 h of abstinence prior to limited access 2BC to water and alcohol in their home cage for 5 test drinking days. Half of the air and CIE-exposed mice were subjected to a 10-min FSS test in a glass cylinder (20 cm diameter × 40 cm high) that was half-filled with 23–25 °C tap water following previously reported methods^43–46^. After removal from the swim test cylinders, mice were hand-dried and placed on a heating pad for 5–10 min. For mice in the home cage drinking studies, mice were given access to alcohol or water 4 h following removal from swim test cylinders. The non-stressed mice remained undisturbed in their home cage. There was 72 h between the last test drinking session and the start of the next cycle of vapor exposure. Body weights were recorded weekly during drinking weeks and daily during cycles of CIE exposure. In mice tested for the ability of 1-1-ethyl-2-benzimidazolinone (1-EBIO) to reduce stress-induced drinking, mice were administered either vehicle (0.5% dimethyl sulfoxide (DMSO) in 0.9% saline) or 10 mg/kg 1-EBIO 30 min prior to alcohol access and approximately 3 h after stress (control) exposure during test week 2. Mice tested for the effects of CIE × FSS on negative affective behaviors (marble burying and NSFT) were subjected to an abbreviated CIE × FSS model, whereby they only received CIE (or air) exposure and FSS (or no stress) but were not given access to alcohol in the home cage. Separate cohorts of mice were used for the marble burying and NSFTs.

### Marble burying test

The marble burying test followed previously described procedures^49^. Testing on this task occurred 12–13 days after CIE exposure and 7 days after FSS. Briefly, mice were placed in standard polycarbonate home cages with ~4 cm of fresh cob bedding for 2 h. They were returned to their home cage and 20 marbles were added to the test cage using a template. Mice were then placed in the test cage for 30 min and the number of buried marbles (>75%) was determined.

### Novelty-suppressed feeding test

Seven-to-8 days after the last FSS (12–13 days post CIE exposure), non-drinking mice were tested on the NSFT in a well-lit room, as described previously^7,10^. Briefly, mice were food deprived for 48 h with a 2-h free-feeding access period from hours 23–25 of deprivation. Mice were allowed to habituate to the testing room for 1 h before testing. At the start of NSFT, mice were then placed in the corner of an open field apparatus (40 × 40 × 40 cm) containing a single food pellet in the center and ~2 cm of fresh cob bedding on the floor. The latency to initiate food consumption was recorded and mice were immediately removed from the apparatus and placed in their home cages with a pre-weighed food pellet on the floor. Mice were allowed to consume the food pellet for 5 min. In separate cohorts, mice were administered (a) vehicle (0.5% DMSO in 0.9% sterile saline; intraperitoneal (i.p.); 10 ml/kg dose volume) or 1-EBIO (2.5–10 mg/kg; Tocris) or (b) vehicle (0.9% saline) or apamin (0.4 mg/kg; Sigma-Aldrich, St Louis, MO) 30 min prior to behavioral testing.

### Long-access alcohol drinking

Before testing in the CIE-FSS drinking model, the ability of 1-EBIO to reduce alcohol intake was established in a long-access 2BC paradigm. In this experiment, mice were given 22 h access to water or alcohol (15% v/v) until a stable baseline of consumption was established across 5 weeks of drinking. Vehicle or 1-EBIO (2.5, 5, and 10) was administered i.p. 30 min prior to alcohol availability, and intake levels were measured at 2, 4, and 22 h. Drug was administered once/week across 3 weeks in a counterbalanced within-subjects design. Non-cumulative drinking data at the different time points were used for analysis.

### Locomotor testing

Locomotor activity was recorded in automated activity chambers (MED Associates, St. Albans, VT). C57BL/6J mice underwent a 10-min habituation session for 3 days followed by three daily 30-min test sessions. Thirty minutes prior to each habituation session, mice received an injection of vehicle. For test sessions, mice received either vehicle or 1-EBIO (2.5 or 10 mg/kg) in a counterbalanced within-subjects design.

### Statistical analysis

A mixed data procedure was used in the statistical software language SAS (SAS Institute Inc., Cary, NC) to analyze all behavioral data following previous methods^18,50^ because of the capacity to model the variance and correlation structure and handle unbalanced repeated-measures data^51^. Behavioral data were nested within mouse and further nested by time or session. Tukey’s test was used for all post hoc analyses of behavioral data, unless otherwise noted. Group size estimates were chosen based on previous behavioral studies and power analyses in drinking mice. All data are reported as mean ± standard error of the mean, and statistical significance was established with an *α* of 0.05. Variances were similar between groups and individual values for each experimental group are reported in the Figures or Supplemental Tables. No outliers were included in the results reported, and technical staff were blinded to experimental groups during behavioral testing.

## Results

### Genetic predictors of anxiety-related phenotypes

**Fig. 1:**
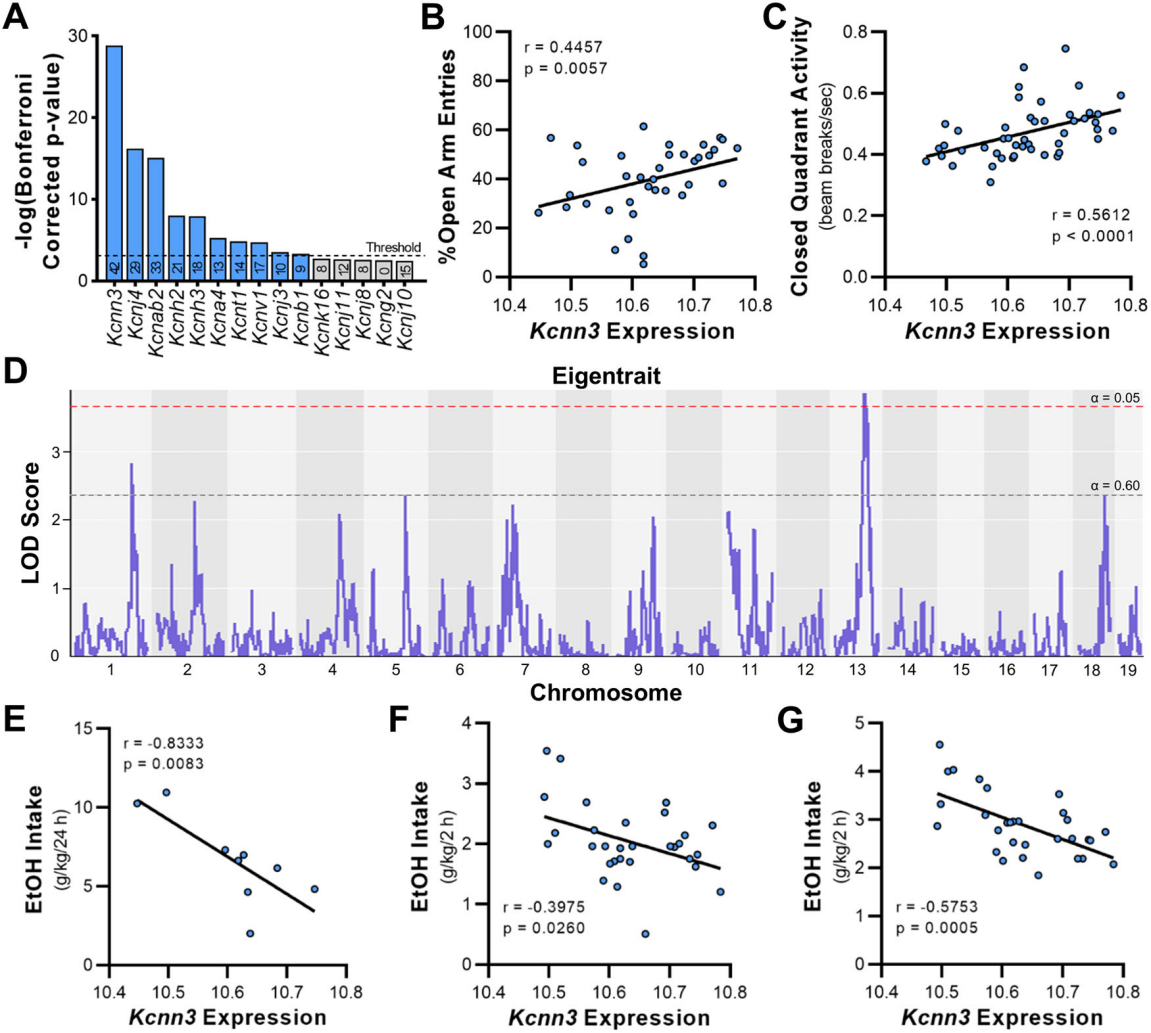
*Kcnn3* enrichment in anxiety-like and activity-based behaviors and correlations with alcohol drinking in BXD RI strains of mice. **A** Enrichment analysis of K^+^ channel genes in the BLA and anxiety-related phenotypes. The number contained within each bar represents the number of record IDs for anxiety- and activity-related phenotypes that are significantly correlated with each gene. **B**, **C** Significant positive correlations between BLA *Kcnn3* expression and open arm entries (GeneNetwork.org Record ID 11714; *n* = 37 strains) and closed quadrant activity (Record ID 12354; *n* = 48 strains). **D** Mapping analysis of the *Kcnn3*-seeded eigentrait variance generated using the seven phenotypes that have correlations with BLA *Kcnn3* expression. Genome-wide significance thresholds are indicated for *α* = 0.05 (LOD) = 3.67; red dashed line) and *α* = 0.6 (LOD) = 2.36; gray dashed line) and are based on 1000 permutations of the data. Representative negative correlations between BLA *Kcnn3* and alcohol consumption in **E** a 24-h access model (3% EtOH v/v in 0.2% saccharin; Record ID 10475; *n* = 9 strains); **F** the baseline phase of the drinking in the dark (DID) model (20% EtOH v/v; Record ID 20264; *n* = 31 strains), and **G** DID drinking following chronic mild stress exposure (Record ID 20288; *n* = 31 strains). The GeneNetwork probe set for BLA *Kcnn3* used for these analyses is Record ID 10493555. *r* and *p* values in **B**, **C**, and **E**–**G** represent Spearman correlation analysis generated in GeneNetwork.

To identify K^+^ channel genes that are associated with anxiety-related phenotypes, we performed an unbiased targeted genetic screen using amygdala K^+^ channel transcript levels and phenotypic data in BXD RI strains of mice. Enrichment analysis identified ten K^+^ channel genes that were over-represented with significant correlations with anxiety-like behaviors in BXD RI strains (Fig. 1A). The top gene, *Kcnn3* (*Chr 3* at 89.52 Mb), encodes a small-conductance, calcium-activated K^+^ (K_Ca_2.3 or SK3) channel. Further examination of *Kcnn3* expression in the amygdala (but not nucleus accumbens—data not shown) revealed a strong eigentrait for anxiety-like and activity-based behaviors (Supplemental Table 1). Representative examples of correlations between BLA *Kcnn3* expression and anxiety-like behavior and activity in BXD RI strains are shown in Fig. 1B, C. We next identified what loci control the variability in the *Kcnn3*-seeded network. Mapping analysis revealed that the *Kcnn3* eigentrait variance is polygenic, as there were genomic regions on *Chr 13* and *Chr 1* containing gene variants associated with eigentrait expression at a genome-wide significance level of *p* < 0.05 and suggestive level of *p* < 0.60, respectively (Fig. 1D). These findings suggest that the *Kcnn3* “locomotor and anxiety” eigentrait is part of a complex phenotype involving genetic variation upstream of *Kcnn3*.

### BLA *Kcnn3* expression and alcohol consumption

We next determined whether *Kcnn3* expression in the BLA also correlated with voluntary drinking in BXD RI strains of mice. As shown in Supplemental Table 2, analysis revealed significant correlations between *Kcnn3* and alcohol consumption in multiple drinking models, the majority of which (15 of 16) were negative correlations. Representative correlations between BLA *Kcnn3* expression and alcohol drinking in standard long-access home cage and drinking in the dark (DID) models are shown in Fig. 1E, F. Notably, many of the significant correlations between *Kcnn3* and binge-like drinking are from mice that were exposed to chronic mild stress (see Supplemental Table 2 and Fig. 1G).

### CIE and FSS effects on behavior

**Fig. 2:**
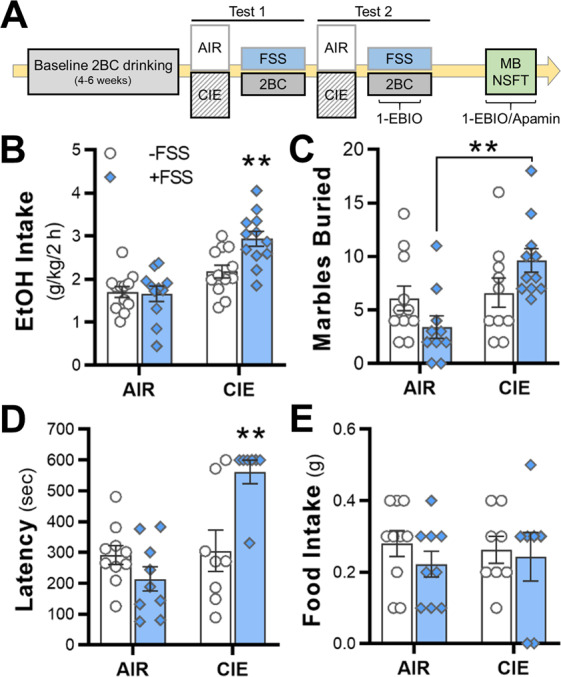
Drinking and negative affective-like behaviors in mice co-exposed to chronic intermittent alcohol (CIE) and forced swim stress (FSS). **A** Schematic of the two-bottle choice (2BC) drinking model in mice exposed to CIE × FSS. Testing on the marble burying (MB) and novelty-suppressed feeding tests (NSFT) occurred during abstinence in non-drinking mice. 1-EBIO or apamin were administered 30 min prior to home cage drinking or the NSFT. **B** Alcohol consumption in mice exposed to CIE and daily FSS 4 h prior to home cage alcohol drinking (*n* = 11–12 mice/group). **C** The number of marbles buried in a 30-min test period (*n* = 10–11 mice/group). **D**, **E** Latency to consume food on the NSFT and post-test food consumption in the home cage (*n* = 7–10 mice/group). **p < 0.01 vs all groups in **B**, **D** and Air vs CIE mice in FSS groups in **C**.

After identifying a top candidate gene that is associated with anxiety-related and alcohol drinking phenotypes, we next tested whether an alcohol-stress interaction model (Fig. 2A) that produces increases in drinking and cognitive deficits also produces negative affective behaviors during abstinence. Consistent with previous studies^43–45^, voluntary alcohol intake was increased in alcohol-dependent mice that were exposed to a 10-min FSS that occurred 4 h prior to the start of the drinking session (Fig. 2B). A two-factor analysis revealed a significant interaction between CIE × FSS exposure during the drinking session in test 2 (*F*_1,43_ = 6.49, *p* = 0.0145; *n* = 11–12 mice/group), and post hoc analysis showed that stressed, alcohol-dependent mice drank more alcohol in 2 h than the mice in the other three treatment groups (*p* < 0.01).

During abstinence from CIE × FSS exposure, two separate cohorts of mice were tested on measures of negative affective behaviors. In the first cohort, mice were tested on the marble burying task. There was a significant interaction (*F*_1,39_ = 5.91, *p* = 0.0197, *n* = 10–11 mice/group; Fig. 2C) with post hoc analysis revealing a difference between stressed mice in the Air and CIE groups. The second cohort of mice was tested on the NSFT. There was a significant interaction (*F*_1,30_ = 13.82, *p* = 0.0008; *n* = 7–10 mice/group; Fig. 2D), and post hoc tests revealed that stressed, alcohol-dependent mice had longer latencies to consume food in comparison with mice in the remaining three groups (*p* < 0.01 vs all groups). Food consumption in the 5 min home cage post-test was similar across all groups (main effects: *F*_1,30_ = 0.77; *p* = 0.387, and *F*_1,30_ < 0.01, *p* = 0.972; Fig. 2E), suggesting that motivation to consume food in the home cage was not affected by CIE, FSS, or their interaction.

### K_Ca_2 channels and NSFT

**Fig. 3:**
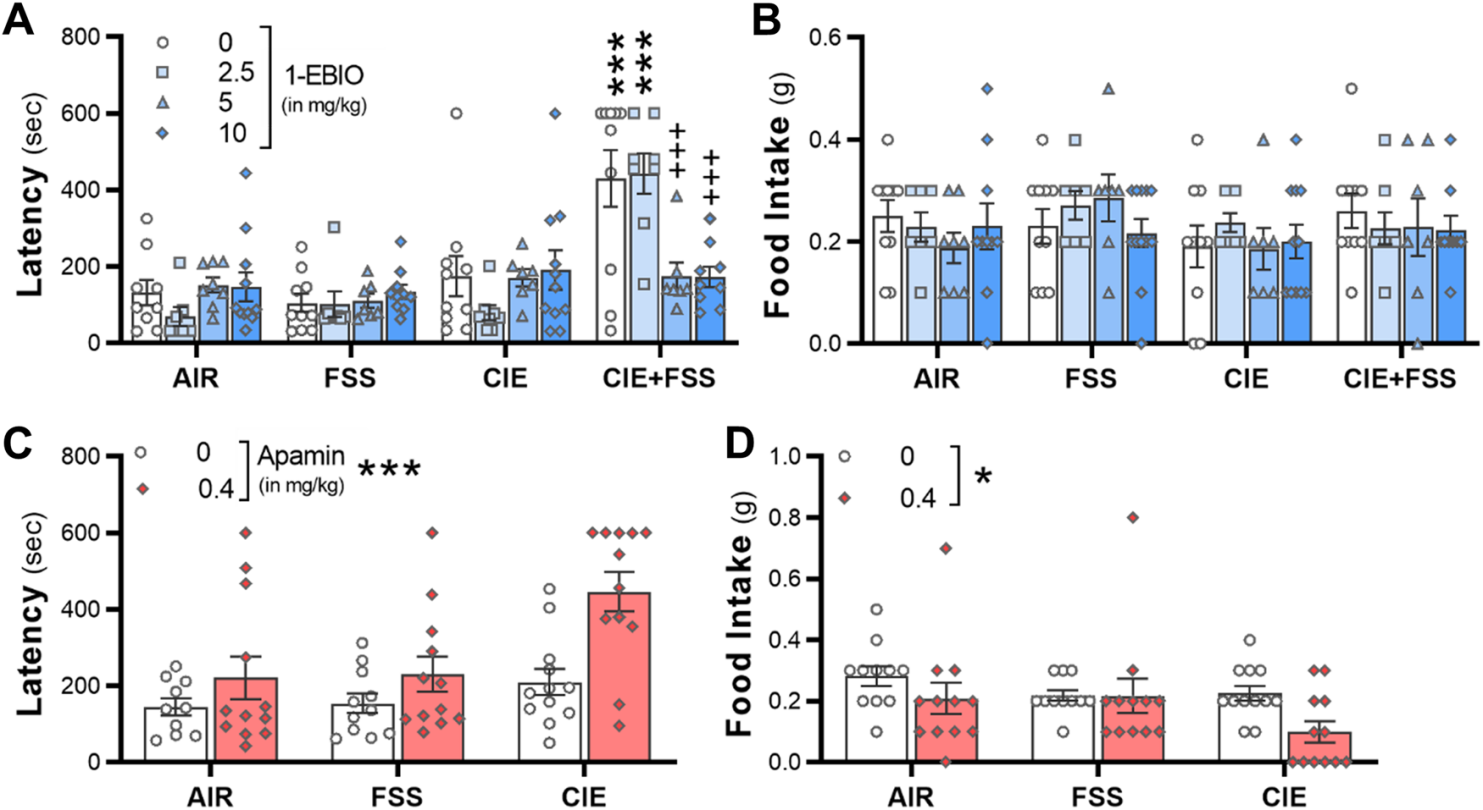
K_Ca_2 channels bidirectionally regulate feeding behavior on the novelty-suppressed feeding test in stressed and alcohol dependent mice. **A**, **B** 1-EBIO significantly reduced latency to feed in stressed, dependent mice, without affecting post-test food consumption (*n* = 7–10 mice/group). **C**, **D** Apamin (0.4 mg/kg, i.p.) significantly increased latency to consume food in the NSFT. Food intake in the home cage following the NSFT was reduced by apamin (*n* = 11–12 mice/group). *p < 0.05, ***p < 0.001 vs vehicle-treated mice. ^+++^*p* < 0.001 vs vehicle and 2.5 mg/kg 1-EBIO in CIE-FSS mice.

Given the strong association between amygdala *Kcnn3* and negative affective and voluntary drinking behaviors, a pharmacological approach was used to determine whether increasing activity of K_Ca_2 channels would reduce latency on the NSFT and excessive drinking in stressed, alcohol-dependent mice. Because of the lack of commercially available ligands that selectively activate K_Ca_2.3 channels, mice were treated systemically with the K_Ca_2.1–2.3 channel activator 1-EBIO (2.5–10 mg/kg)^52^ or vehicle 30 min prior to the NSFT. A three-factor analysis revealed a significant interaction (*F*_3,123_ = 4.25, *p* = 0.007, *n* = 7–11 mice/group; Fig. 3A). There were also significant simple interactions for vehicle (*F*_1,123_ = 13.35, *p* = 0.0004) and 2.5 (*F*_1,123_ = 14.23, *p* = 0.0003) but not for 5 (*F*_1,123_ = 0.25, *p* = 0.615) or 10 mg/kg (*F*_1,123_ < 0.01, *p* = 0.946) doses of 1-EBIO. Post hoc analyses revealed that stressed, alcohol-dependent mice treated with vehicle or 2.5 mg/kg 1-EBIO had longer latencies to consume food during the NSFT compared with vehicle-treated mice in the three other groups, as well as compared with stressed, alcohol-dependent mice treated with 5 or 10 mg/kg 1-EBIO. Food consumption in the 5 min post-test was similar across all treatment groups (*F*_3,123_ = 0.01; *p* = 0.391; Fig. 3B). Simple interactions for treatment × group, treatment × dose, and group × dose were also not significant (*p* ≥ 0.612).

To complement the previous findings with 1-EBIO, we tested whether K_Ca_2 channels have bidirectional control over NSFT behavior by treating mice in the CIE and FSS groups with the K_Ca_2.2–2.3 channel allosteric inhibitor apamin. There were significant main effects for treatment (*F*_2,64_ = 6.78; *p* = 0.0021) and drug (*F*_1,64_ = 12.18; *p* = 0.0009), but the interaction did not reach statistical significance (*F*_2,64_ = 2.67; *p* = 0.0772; *n* = 11–12 mice/group; Fig. 3C). Post hoc analysis showed that mice pretreated with 0.4 mg/kg apamin had increased latency to feed compared with vehicle and that CIE-exposed mice had longer latencies to feed than control and FSS mice (*p* < 0.05). There was also a main effect of drug for food intake in the post-NSFT home cage test (*F*_1,64_ = 4.28; *p* = 0.0426; Fig. 3D), revealing a reduction in amount of chow consumed in 5 min in the apamin-treated mice, which is consistent with a previous study in mice^53^.

### 1-EBIO reduces alcohol drinking

**Fig. 4:**
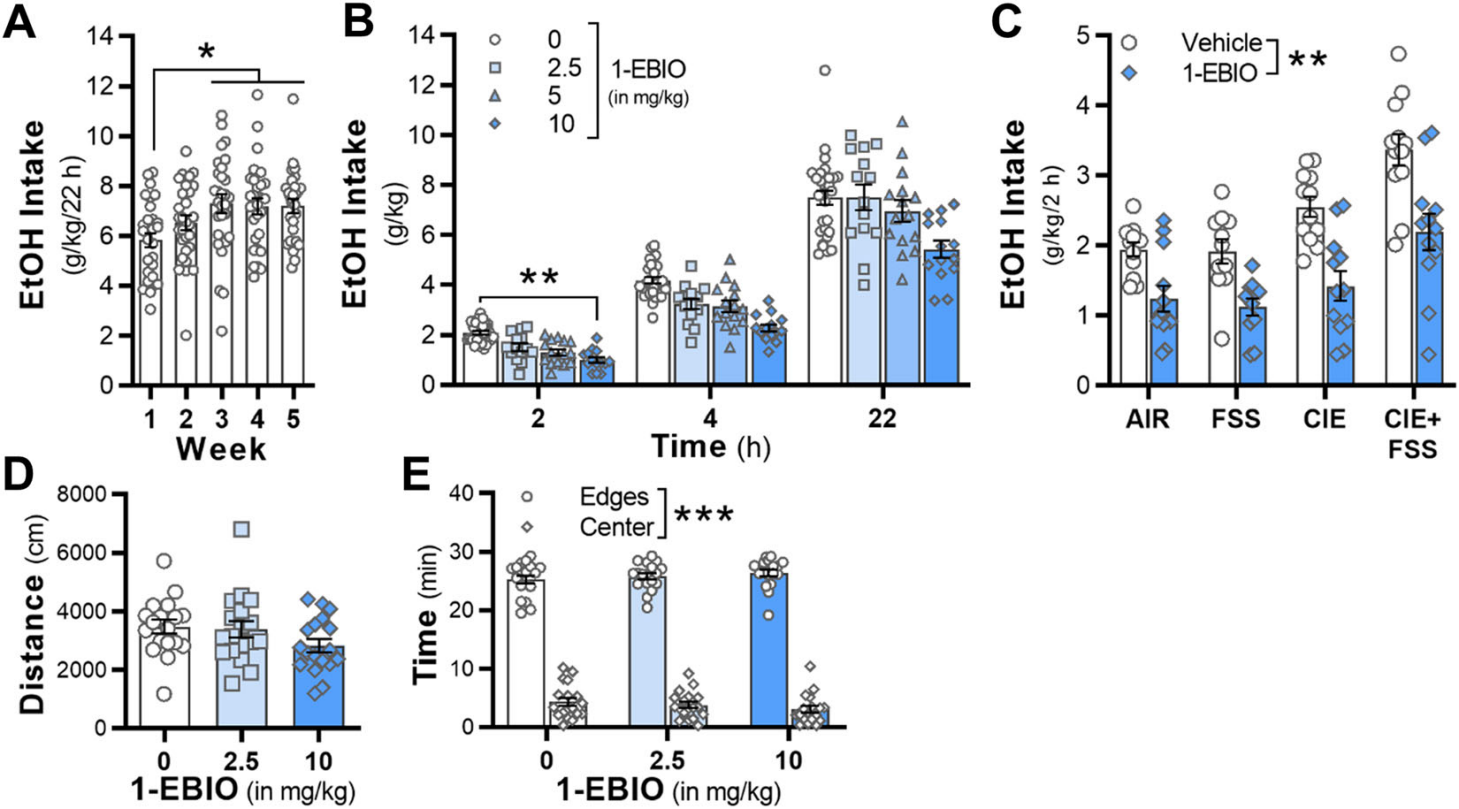
Positive modulation of K_Ca_2 channels reduced alcohol intake. **A** Mice escalate voluntary alcohol drinking when given 22 h of access to ethanol (*n* = 29 mice). 1-EBIO significantly reduced alcohol drinking in the **B** long- and **C** short-access home cage (*n* = 11–12/group) models regardless of treatment group. **D**, **E** 1-EBIO did not reduce overall distance traveled or time spent in the center or near the edges of an open field apparatus (*n* = 18 mice). *p < 0.05, weeks 3, 4, and 5 vs week 1. **p < 0.01, vehicle vs 1-EBIO. ***p < 0.001, edges vs center.

Because 1-EBIO reduced alcohol-seeking behaviors in rats^54,55^, we also wanted to test the ability of 1-EBIO to reduce excessive drinking in another species. C57BL/6J mice were given daily access to 22 h of alcohol (15%, v/v) or water. In this model, mice increased their drinking in weeks 3–5 in comparison with drinking amounts in the first week of access to alcohol (*F*_4,28_ = 5.22, *p* = 0.003, *n* = 29 mice; post hoc, *p* < 0.05 vs week 1; Fig. 4A). There was a significant interaction with time and drug dose (*F*_6,80_ = 3.66, *p* = 0.0029; Fig. 4B). Post hoc analyses revealed that 10 mg/kg 1-EBIO significantly reduced drinking at the 2 h time point (*p* = 0.0151) in comparison with vehicle treatment.

Mice exposed to the CIE × FSS paradigm were treated with vehicle and 10 mg/kg 1-EBIO in a within-subjects design across two drinking sessions during test 2. While the three-factor interaction was not significant (*F*_1,44_ = 0.02, *p* = 0.894; *n* = 11–12/group), there was a significant main effect of drug treatment (*F*_1,44_ = 94.28, *p* < 0.0001) and a significant two-factor interaction for Air/CIE × drug treatment (*F*_1,44_ = 4.28, *p* = 0.044). Further analysis of the simple effects revealed that 1-EBIO significantly reduced alcohol drinking in Air and CIE-exposed mice, regardless of stress history (*F*_1,44_ = 9.48, *p* < 0.01). The effect of 1-EBIO was not due to locomotor deficits, as overall locomotor behavior in an open field test was similar across vehicle and dose (*F*_2,28_ = 2.38, *p* = 0.111, *n* = 18 mice; Fig. 4D), consistent with previous reports in mice^56,57^. While mice spent more time near the walls than the center of the open field arena (*F*_1,17_ = 2158.17, *p* < 0.0001), there was no drug effect on time spent in center or edges (*F*_2,34_ = 0.01, *p* = 0.986; Fig. 4E).

## Discussion

In this study, we first used a bioinformatics discovery tool to identify K^+^ channel genes in the BLA of genetically diverse mice that correlated with both anxiety-like and high alcohol drinking phenotypes. Next, we demonstrated that a model for the study of alcohol–stress interactions that has been shown to increase alcohol drinking, cortical metaplasticity, and cognitive impairments^43–45,58,59^ also produced negative affective behaviors in C57BL/6J mice. We then provided evidence for pharmacological validation of the top gene (i.e., *Kcnn3*) in the CIE × FSS model on negative affective behaviors and stress-induced increases in alcohol drinking. Specifically, positive modulation of K_Ca_2 channels reduced alcohol drinking in all mice and reversed the hypophagia during the NSFT in stressed, dependent mice. By identifying a role for *Kcnn3* in regulating excessive drinking and negative affective behaviors in stressed, dependent mice, these preclinical studies demonstrate that K_Ca_2 channels are a potential pharmacogenetic target to treat individuals with AUD and may be particularly effective at treating those with comorbid mood disorders.

Using an exploratory analysis targeting BLA K^+^ channel genes of BXD RI strains, we identified a *Kcnn3* eigentrait for anxiety- and activity-related behavioral traits. Consistent with these results, adult mice with conditional knockout of *Kcnn3* displayed maladaptive coping behaviors on the forced swim and tail suspension tests^27^. In addition to the genetic evidence for *Kcnn3* and anxiety-related behaviors, BLA *Kcnn3* expression was also negatively correlated with alcohol drinking traits in BXD RI strains. Consistent with the negative relationship between BLA *Kcnn3* expression and alcohol drinking that was identified in the current manuscript, we previously reported that *Kcnn3* expression in the nucleus accumbens of BXD RI strains negatively correlated with intake and preference in multiple models of voluntary drinking, including the DID model that produces binge-like alcohol consumption and the CIE-induced escalation of drinking model^39,40^. The BLA correlations were observed in drinking paradigms using a range of alcohol concentrations (3–20%, v/v) and access times (2–24 h), and nearly half of the intake traits are from mice that were exposed to a regimen of chronic mild stress in the DID model. In addition to the genetic evidence for *Kcnn3* and anxiety-related and alcohol drinking behaviors, we found a complex and polygenic trait architecture through genetic mapping of the *Kcnn3* “anxiety” eigentrait with evidence of regulation by loci on *Chr 1* and *13*. The *Chr 1* locus contained single-nucleotide polymorphisms in the *Cacna1e* gene that encodes R-type Ca^2+^ channels. Interestingly, R-type Ca^2+^ channels functionally couple with K_Ca_2 channels^60,61^, and *Cacna1e* knockout mice show anxiety-like behaviors in an open field test^62,63^. Thus the results from our exploratory bioinformatics analysis add to a growing literature implicating *Kcnn3* in anxiety-related and alcohol drinking behaviors and identified a top target that was further explored in a chronic alcohol and stress exposure model.

After replicating previous findings of higher alcohol intake in mice exposed to CIE and FSS, we tested mice on two different tasks that model negative affective behaviors. While we did not observe a stress–alcohol interaction on the marble burying test similar to a previous study^58^, there was a robust behavioral deficit on the latency to consume food in the NSFT in stressed, alcohol-dependent mice. The majority of mice (65%) in the CIE-FSS treatment group did not attempt to feed in the novel environment but showed normal feeding behavior when tested in their home cage. Comparatively, nearly 95% of the mice that were treated with either FSS or CIE consumed food during the NSFT. The absence of negative affective behaviors in CIE-exposed mice is in contrast to reports from recent studies^49,64,65^. The reason for the discrepancy likely relates to the comparatively shorter duration of alcohol exposure in our study where mice were exposed to two cycles of CIE exposure with a week in between cycles. Similar to CIE exposure, repeated FSS exposure alone did not produce negative affective behaviors in our study. This truncated CIE exposure model was designed specifically to explore the facilitation of aberrant behaviors in CIE-FSS mice. With additional CIE cycles and FSS tests, we would expect to see escalated drinking and negative affective behaviors emerge in mice exposed to CIE or FSS alone. Together, these findings suggest that that there is a complex interaction between stress and alcohol that facilitated the escalation of alcohol intake and the development of maladaptive behaviors on some but not all tasks that model negative affective behaviors.

Using pharmacology as a translational tool to validate the bioinformatics analysis, we found that enhancing K_Ca_2 channel activity decreased the latency to feed on the NSFT in stressed, dependent mice. We also demonstrated that blocking K_Ca_2 channels with apamin increased latency to feed on the NSFT in control mice, as well as mice that were exposed to chronic FSS or CIE, recapitulating the interactive effects of CIE and FSS and providing evidence for bidirectional control of K_Ca_2 channels over negative affective behaviors. In addition to reducing hypophagia in stressed, dependent mice, we also showed that 1-EBIO attenuated alcohol drinking in short- and long-access models. In the 22 h drinking model, the reduction in drinking by 1-EBIO was only observed at 2 h time point, although intake remained below values in vehicle-treated mice at the later time points. While the half-life of 1-EBIO is not reported in the literature, the relatively short-lived reduction in alcohol intake suggests that 1-EBIO may be rapidly metabolized in adult mice. In the alcohol–stress co-exposure model, 1-EBIO decreased drinking in all groups, including the control, non-stressed mice, without affecting ambulatory activity. These findings with 1-EBIO are consistent with previous evidence for reductions in drinking using K_Ca_2 channel-positive modulators across multiple models of voluntary intake and seeking behaviors in rodents^54,55,66^. Similar to our results with apamin in the NSFT, we previously reported an increase in alcohol drinking when apamin was microinfused into the accumbens of non-dependent C57BL/6J mice^40^. Together, these converging lines of evidence suggest that alcohol intake is under the bidirectional control of the *Kcnn3*/K_Ca_2 channels in genetically diverse mice and that K_Ca_2 channels are a target to reduce moderate and excessive amounts of alcohol drinking.

To our knowledge, this is the first study to explore *Kcnn3*/K_Ca_2 channels at the intersection of stress and alcohol on both drinking and negative affective behaviors. However, there is evidence from previous studies implementing K_Ca_2 channels in behaviors related to alcohol use or mood disorders. Chronic alcohol exposure in adolescent and adult rodents reduced K_Ca_2 channel currents and protein expression in cortical and subcortical regions^40,54,67–69^. 1-EBIO microinfusion into the accumbens shell attenuated anxiety-like behavior in alcohol-exposed adolescent rats^70^. Similarly, expression and function of BLA K_Ca_2 channels were decreased in socially isolated adolescent rats and chronically stressed adult mice^26,71^, and pharmacologically enhancing K_Ca_2 channel activity systemically or directly in the BLA reduced negative affective behaviors^57,71^. Consistent with this, a previous study demonstrated that K_Ca_2.2 overexpression in the BLA decreased anxiety-like behaviors in non-stressed mice^72^, and overexpression of K_Ca_2.2 channel in ventral hippocampal-projecting BLA neurons prevented anxiety-like behaviors in stressed mice^26^. In postmortem samples, *KCNN2* expression was reduced in the BLA and frontal cortex of individuals with AUD^73^, and *KCNN3* was upregulated in the striatum in individuals with bipolar disorder^74^. Although these findings demonstrate that the relationship between alcohol, stress, and K_Ca_2 channels is complex and depends on brain region and age of stress or alcohol exposure, our findings add to a growing literature that K_Ca_2 channels are a crucial ion channel family that is important for driving interactions of stress- and alcohol-related behaviors.

Despite the converging genetic and pharmacological evidence that K_Ca_2 channels control alcohol–stress interactions, there are a few limitations to the current study that should be considered. First, although genetic data suggest the BLA as a locus of altered K_Ca_2 channel dysfunction, adaptations in K_Ca_2 channels in the BLA of mice treated with the CIE-FSS model were not directly assessed, nor did we specifically manipulate BLA K_Ca_2 channels in our pharmacological experiments. Rather, a systemic pharmacological approach was selected to provide translationally relevant findings using the CIE-FSS mouse model for the study of alcohol–stress behavioral outcomes. Second, while 1-EBIO and apamin both have actions on K_Ca_2.3 channels^75,76^, they also influence other K_Ca_2 channel subtypes and may have off-target actions on other ion channels^77,78^. However, previous studies have demonstrated that apamin reverses 1-EBIO-induced reductions in neural function^52,79^, suggesting that the bidirectional changes in drinking and negative affective behaviors by these compounds is mediated primarily through actions on K_Ca_2 channels. Lastly, to our knowledge, compounds that selectively inhibit or activate K_Ca_2.3 channels are not commercially available.

In summary, the bioinformatics discovery approach and pharmacological validation results demonstrate a role for *Kcnn3*/K_Ca_2 channels in mediating negative affective behaviors and alcohol drinking in mice. Our findings also demonstrate robust behavioral effects of stress–alcohol interactions on alcohol drinking and NSFT that can be reduced by activation of K_Ca_2 channels. Thus K_Ca_2 channel-positive modulators appear to regulate alcohol consumption, in general, and might affect some aspects of affective disorders precipitated by alcohol–stress interactions making it an appealing targeted approach to treating comorbid anxiety and AUDs. Ultimately, clinical trials will be necessary to test the efficacy of K_Ca_2 channel-positive modulators as a possible pharmacogenetic target for treating individuals with comorbid mood and AUDs, especially if selective K_Ca_2.3 channel ligands become available.

## Supplementary information

Supplemental Tables
