# Supplementary material for: Bioinformatics identification and pharmacological validation of Kcnn3/KCa2 channels as a mediator of negative affective behaviors and excessive alcohol drinking in mice
Source: Transl Psychiatry. 2020 Nov 27;10:414. doi: 10.1038/s41398-020-01099-4 (PMC7699620; doi:10.1038/s41398-020-01099-4)
Supplement: Supplementary file 1 — Supplemental Tables [file 41398_2020_1099_MOESM1_ESM.docx]

**Supplemental Table 1 -** Anxiety-like behavior and activity eigentrait computed from seven behavioral traits. All data are publically available on genenetwork.org. *p* and *r* values were obtained from Spearman correlation analysis performed in GeneNetwork.

| **Record ID** | **Phenotype** | **Authors** | **Max LRS Location** | **r** | ***n*** | ***p*** |
| --- | --- | --- | --- | --- | --- | --- |
| 11398 | Fear conditioning response, activity during second tone-shock pairing for males [units] | Philip VM, Ansah TA, Blaha CD, Cook MN, Hamre KM, Lariviere WR, Matthews DB, Mittleman G, Goldowitz D, Chesler EJ | Chr13: 83.736987 | -0.5585 | 38 | 0.00019 |
| 11410 | Open field behavior, locomotion from 5-10 min for males [cm] | Philip VM, Ansah TA, Blaha CD, Cook MN, Hamre KM, Lariviere WR, Matthews DB, Mittleman G, Goldowitz D, Chesler EJ | ChrX: 3.231738 | -0.4585 | 38 | 0.00338 |
| 11649 | Fear conditioning response, activity in altered context for females [units] | Philip VM, Ansah TA, Blaha CD, Cook MN, Hamre KM, Lariviere WR, Matthews DB, Mittleman G, Goldowitz D, Chesler EJ | Chr13: 78.232552 | -0.5349 | 35 | 0.00073 |
| 11714 | Anxiety assay, untreated baseline, percent entries into open arms of an elevated plus maze for females [%] | Philip VM, Ansah TA, Blaha CD, Cook MN, Hamre KM, Lariviere WR, Matthews DB, Mittleman G, Goldowitz D, Chesler EJ | Chr11: 120.956900 | 0.4459 | 37 | 0.00517 |
| 12354 | Anxiety assay, baseline untreated control (BASE group), activity in closed quadrants using an elevated zero maze in 60 to 120-day-old males only during 10 min [beam breaks/sec] | Melloni Cook, Lu Lu, Rob Williams | Chr4: 105.245023 | 0.5516 | 48 | 3.13E-05 |
| 12420 | Anxiety assay, saline treated [0.18 ml/kg i.p.] (NOS group), activity in closed quadrants using an elevated zero maze in 60 to 120-day-old males and females during last 5 min [n beam breaks] | Melloni Cook, Lu Lu, Rob Williams | Chr1: 153.969506 | -0.5415 | 46 | 7.02E-05 |
| 12480 | Anxiety assay, restraint stress [15 min] + saline treated [.018 ml/kg i.p.] (RSS group), activity in closed quadrants using an elevated zero maze in 60 to 120-day-old males and females during last 5 min [n beam breaks] | Melloni Cook, Lu Lu, Rob Williams | Chr1: 153.969506 | -0.5226 | 48 | 0.0001 |

**Supplemental Table 2** – Correlations between *Kcnn3* expression in the basolateral amygdala (INIA Amygdala Cohort Affy MoGene 1.0 ST (Mar11) RMA Database; Record ID 10493555) and voluntary alcohol (ethanol) drinking in male and female BXD RI strains of mice. DID = drinking in the dark. *p* and *r* values were obtained from Spearman correlation analysis performed in GeneNetwork.

| **Record ID** | **Phenotype** | **Authors** | **Year** | **Max LRS Location (Chr and Mb)** | **r** | **N** | **P** |
| --- | --- | --- | --- | --- | --- | --- | --- |
| 20288 | Ethanol consumption of 20% v/v EtOH using the DID method, 2 h access on day 2 week 1 of period 2 (P2, chronic mild stress period), in chronic mild stress 4-6 month old females [g/kg] | Jones BC, Lu L, Mormède P, Terenina E, Mulligan MK, Cavigelli SA, Zhao W, Williams RW | 2018 | Chr7: 144.447601 | -0.5753 | 31 | 0.00052 |
| 20293 | Ethanol consumption of 20% v/v EtOH using the DID method, 2 h access on day 2 week 2 of period 2 (P2, chronic mild stress period), in chronic mild stress 4-6 month old females [g/kg] | Jones BC, Lu L, Mormède P, Terenina E, Mulligan MK, Cavigelli SA, Zhao W, Williams RW | 2018 | Chr6: 90.017712 | -0.5275 | 31 | 0.00191 |
| 10475 | Ethanol (3%, g/kg in 0.2% saccharin and tap water) consumption using a two-bottle choice test vs. tap water, mean of day 2 and day 4 of a 4-day 24-h access period [g/kg/day] | Phillips TJ, Crabbe JC, Metten P, Belknap JK | 1994 | Chr4: 155.502574 | -0.8333 | 9 | 0.00332 |
| 10477 | Ethanol (10%, g/kg in 0.2% saccharin and tap water) consumption using a two-bottle choice test offered vs. tap water, mean of day 2 and day 4 of a 4-day 24-h access period [g/kg/day] | Phillips TJ, Crabbe JC, Metten P, Belknap JK | 1994 | Chr3: 141.733523 | -0.8167 | 9 | 0.00497 |
| 20292 | Ethanol consumption (20% v/v EtOH) using the DID method, 2 h access on day 1 week 2 of period 2 (P2, chronic mild stress period), in chronic mild stress 4-6 month old females [g/kg] | Jones BC, Lu L, Mormède P, Terenina E, Mulligan MK, Cavigelli SA, Zhao W, Williams RW | 2018 | Chr2: 173.244458 | -0.4483 | 31 | 0.01067 |
| 20312 | Ethanol consumption (20% v/v EtOH) using the DID method, 2 h access on day 1 Week 6 of period 2 (P2, chronic mild stress period), in chronic mild stress 4-6 month old females [g/kg] | Jones BC, Lu L, Mormède P, Terenina E, Mulligan MK, Cavigelli SA, Zhao W, Williams RW | 2018 | Chr11: 120.956900 | -0.439 | 31 | 0.01269 |
| 20264 | Ethanol consumption (20% v/v EtOH) using the DID method, 2 h access on day 1 week 2 of period 1 (P1, baseline), in chronic mild stress females [g/kg] | Jones BC, Lu L, Mormède P, Terenina E, Mulligan MK, Cavigelli SA, Zhao W, Williams RW | 2018 | Chr5: 135.879195 | -0.3975 | 31 | 0.02602 |
| 20282 | Ethanol consumption (20% v/v EtOH) using the DID method, 4 h access on day 4 week 5 of period 1 (P1, baseline), in chronic mild stress females [g/kg] | Jones BC, Lu L, Mormède P, Terenina E, Mulligan MK, Cavigelli SA, Zhao W, Williams RW | 2018 | Chr1: 167.194662 | -0.3908 | 31 | 0.02895 |
| 20299 | Ethanol consumption (20% v/v EtOH) using the DID method, 2 h access on day 3 week 3 of period 2 (P2, chronic mild stress period), in chronic mild stress 4-6 month old females [g/kg] | Jones BC, Lu L, Mormède P, Terenina E, Mulligan MK, Cavigelli SA, Zhao W, Williams RW | 2018 | Chr8: 7.885228 | -0.3914 | 30 | 0.03167 |
| 20329 | Ethanol consumption (20% v/v EtOH) using the DID method, 2 h access on day 3 week 1 of period 3 (P3, post chronic mild stress), in chronic mild stress 4-6 month old females [g/kg] | Jones BC, Lu L, Mormède P, Terenina E, Mulligan MK, Cavigelli SA, Zhao W, Williams RW | 2018 | Chr9: 81.020335 | -0.3753 | 31 | 0.0368 |
| 20319 | Ethanol consumption (20% v/v EtOH) using the DID method, 2 h access on day 3 week 7 of period 2 (P2, chronic mild stress period), in chronic mild stress 4-6 month old females [g/kg] | Jones BC, Lu L, Mormède P, Terenina E, Mulligan MK, Cavigelli SA, Zhao W, Williams RW | 2018 | Chr9: 85.118181 | -0.3721 | 31 | 0.03863 |
| 13566 | Ethanol consumption (20% v/v EtOH) using DID method (4 h access on day 4 of DID) in females, baseline in chronic mild (CMS) stress group 1 week before start of 7 weeks of CMS (Phase 1) [g/kg] | Jones BC, Lu Lu, Williams RW | 2011 | Chr3: 121.852558 | 0.6205 | 11 | 0.0401 |
| 20284 | Ethanol consumption (20% v/v EtOH) using the DID method, average EtOH consumption for all 4 h in period 1 (P1, baseline), in chronic mild stress 4-6 month old females [g/kg] | Jones BC, Lu L, Mormède P, Terenina E, Mulligan MK, Cavigelli SA, Zhao W, Williams RW | 2018 | Chr1: 167.194662 | -0.3691 | 31 | 0.04041 |
| 10582 | Ethanol (10% in water po) consumption over 24 h for males [g/kg] | Rodriguez LA, Plomin R, Blizard DA, Jones BC, McClearn GE | 1994 | Chr7: 104.149021 | -0.6182 | 11 | 0.04112 |
| 20327 | Ethanol consumption (20% v/v EtOH) using the DID method, 2 h access on day 1 week 1 of period 3 (P3, post chronic mild stress), in chronic mild stress 4-6 month old females [g/kg] | Jones BC, Lu L, Mormède P, Terenina E, Mulligan MK, Cavigelli SA, Zhao W, Williams RW | 2018 | Chr2: 159.228417 | -0.3678 | 31 | 0.04114 |
| 20277 | Ethanol consumption (20% v/v EtOH) using the DID method, 4 h access on day 4 week 4 of period 1 (P1, baseline), in chronic mild stress females [g/kg] | Jones BC, Lu L, Mormède P, Terenina E, Mulligan MK, Cavigelli SA, Zhao W, Williams RW | 2018 | ChrX: 158.300348 | -0.3561 | 31 | 0.04873 |
